# Hemodynamic changes for half cover left subclavian artery ostium during thoracic endovascular aortic repair

**DOI:** 10.3389/fsurg.2024.1399230

**Published:** 2024-08-08

**Authors:** Xiaowei Li, Xiaoming Yuan, Zan Wen, Minghua An, Wei Bi

**Affiliations:** ^1^ Department of Vascular Surgery, The Second Hospital of Hebei Medical University, Shijiazhuang, Hebei, China; ^2^ Department of Cardiovascular Surgery, The First Hospital of Qinhuangdao, Qinhuangdao, Hebei, China; ^3^ Department of Mechatronic Control Engineering, Yanshan University, Qinhuangdao, Hebei, China; ^4^ Department of Ultrasound Medicine, The Haigang Hospital of Qinhuangdao, Qinhuangdao, Hebei, China

**Keywords:** thoracic endovascular aortic repair, endograft, half coverage, left subclavian artery, computational fluid dynamics

## Abstract

**Purpose:**

Some clinicians use endografts to cover half the left subclavian artery (LSA) ostium to cure some cases with insufficient proximal landing zone (PLZ) in thoracic endovascular aortic repair (TEVAR) treatment. We used computational fluid dynamics (CFD) to study the hemodynamic changes in the LSA because they may cause acute thrombosis or arteriosclerosis.

**Methods:**

The digital model of the aortic arch was established and named model A, which only included the supraarch branch of the LSA. By directly covering half of the LSA ostium, which was named as model B. All established models were imported into the Gambit grid division software for grid division and were subsequently imported into the Fluent software for hemodynamic numerical simulation and calculation to analyze the related changes in LSA hemodynamic parameters after stent implantation.

**Results:**

Under the same aortic inlet flow, in model B, the local blood flow velocity of the LSA ostium increased and the whole blood flow velocity at the distal end decreased. The average wall shear stress (WSS) of the LSA was significantly decreased. Meanwhile there was an obvious turbulent flow in the LSA lumen, and the related blood flow state was disordered.

**Conclusion:**

CFD research confirmed that the implantation of an endograft covering half the LSA ostium can cause obvious hemodynamic changes, which is likely to cause a long-term arteriosclerosis or acute thrombosis of the LSA, finally increasing the risk of stroke. Once this operation is performed in some specific clinical cases for simplicity and economy, it seems that we should actively antiplatelet and follow up regularly.

## Introduction

Thoracic endovascular aortic repair (TEVAR), as a lowly invasive surgical technique, has been rapidly developed and greatly reduced the mortality rate. Currently, it has been applied and optimized for the treatment of descending aorta lesions (1). A sufficient and intact proximal landing zone (PLZ) is a necessary anatomical condition for TEVAR and avoidance of the type I endoleak. As such, according to routine requirements, the PLZ should not be less than 15 mm (2, 3). Related research shows that, due to anatomical factors, the primary disease site of the descending aorta in about 40% of patients is close to the LSA (4, 5). Due to the shape of the aortic arch, the distance between partial breaches is less than 15 mm (6). In order to reach the effective anchorage distance, in this study, TEVAR has partially covered the left subclavian artery (LSA) ostium. Some scholars hypothesize that it is safe for some patients to cover the LSA without any revascularisation (7). Nevertheless, completely covering the LSA origin without revascularization may decrease the blood supply of the left upper limb, even increase the risk of paraplegia in some patients, such as those without a dominant or balanced right vertebral artery. As such, most scholars actively recommend LSA revascularisation (8, 9). Therefore, many approaches for the revascularisation of the LSA were developed. Clinicians should provide an individualized treatment according to the characteristics of the patient. Importantly, each operation has its own advantages and limitations (10, 11). However, regardless of the treatment method, it will change the morphological anatomy and hemodynamics of the LSA. For example, some scholars have previously studied the hemodynamics of chimney revascularisation, and proposed that different LSA stent placement positions and directions could produce different results (12). Since hemodynamic factors are the key determinants of atherosclerosis (13, 14), the long-term patency of the LSA in such operations has gradually become the focus of clinical attention.

In the early stage of endovascular treatment or under specific emergency conditions, clinicians extended the PLZ by partially covering the ostium of the LSA to reserve part of the blood supply of the LSA and vertebral artery (15, 16). However, there are few studies on the hemodynamic changes of the LSA using this surgical method, and Some researches are only based on clinical follow-up, which is greatly influenced by the individual factors of patients (heart rate, blood pressure, basic diseases, etc.) (17).

With the rapid development of computational fluid dynamics (CFD), the parameters of intra-arterial hemodynamics can be quickly evaluated. These parameters become a reliable tool to understand hemodynamics, pathological vascular disease progress and the performance of medical equipment (18, 19). Moreover, they can avoid the drawbacks of limited prospective research in clinical trials, time-consuming procedures, high costs, and ethical problems (20). Therefore, in the present work, we studied the hemodynamic changes of the LSA using an endograft covering half the ostium of the LSA. In order to provide an valuable reference for clinical treatment, CFD was applied to analyze the changes in common indices, such as the blood flow field, the blood velocity distribution, and the blood pressure on the arterial wall in the LSA after the implantation of endografts (21).

## Materials and methods

### Test software

The Solidworks 2019 modeling software was adopted in this study. The meshing was divided into the Ansys19.2 Mesh module, and the numerical simulation software used was Fluent19.2.

#### Establishment of a three-dimensional digital model of the aortic

**Figure 1:**
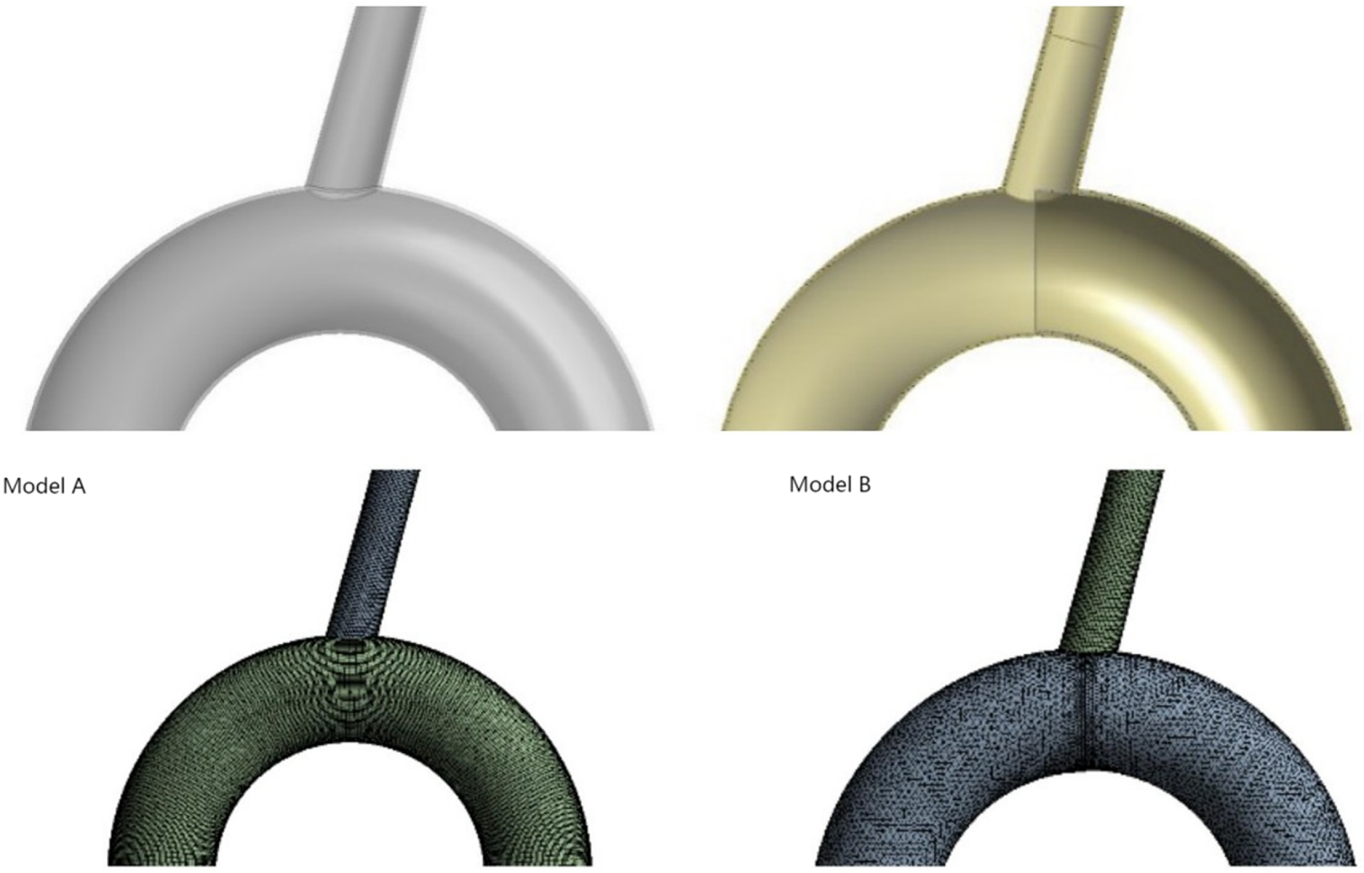
Model diagram of model A and model B (simulated the implantation of endograft stent in aorta covering half of the ostium of LSA). Diagram of grid division in model A and model B after runner extraction was shown in lower part.

In the process of modeling, we try to use MIMICS (Mimics 25, Materialise, Leuven, Belgium) to reconstruct the personalized aortic model. However, the stent is an ideal model, which can not closely fit the aortic wall, which will seriously affect the calculation of computational fluid dynamics. Because the main concern on our study was the influence of stent implantation on the blood flow of the LSA, we used modeling software to simulate the ideal model of the aorta and the LSA. This choice has the advantages of excluding specific errors caused by different individual factors and reducing the number of mesh generation steps. So a three-dimensional digital model including the aortic arch and the LSA branch was established, based on the data of the normal human aortic anatomy (22). The diameter of the aortic arch was 35 mm, the length was 200 mm, the radian was 180 degrees, and the diameter of the LSA branch was 12 mm. This aortic arch model diagram was named model A. Using the Solidworks software, the LSA ostium was half covered with a die as the same thickness as the vascular wall in order to simulate the digital model of endograft to cover half the LSA ostium. Because the bare keel at the front end of the endograft was thin and in a reduced number, it was disregarded. This model was named model B. The flow channel of the blood vessel model was extracted, and the established models were imported into Fluent for mesh generation. The grid number of model A and model B were 208701 and 192911. We show the overall schematic diagrams of the grid division of model A and model B respectively (All process is shown in Figure 1).

### Numerical simulation

#### Governing equations and boundary conditions

In this work, the following assumptions were made: the blood in the aorta behaves as an incompressible Newtonian viscous fluid, the blood flow is laminar according to the Reynolds number; the blood vessel wall is rigid and has no slip (23); the blood flow velocity at the wall surface is 0 m/s; the outlet of the LSA and descending aorta is defined as a free outflow; the pressure is 0 Pa. The blood flow satisfies the Navier-Stokes equation, in which the blood density (*ρ*) is 1,050 kg/m^3^ and the blood viscosity coefficient is *μ*. 0.0035 Pa·s, the number of iterations is 500, the time step is 0.001 s, and the maximum number of iterations is 50 (24).

$$\begin{array}{c} \nabla \cdot u=0\\ \rho \left(\frac{\partial u}{\partial t}+u\cdot \nabla u\right)=-\nabla p+\mu \nabla^{2}u \end{array}$$

#### Numerical simulation

Use FLUENT ENT19.2 computational fluid dynamics software to calculate the change in blood flow velocity. In this study, we assume that the arterial peak inlet velocity is 0.5 m/s. Two cardiac cycles, each of which is 0.5 s, were calculated. When using Fluent to calculate transient step size, it is necessary to set the appropriate time step size, boundary conditions and numerical solutions according to specific problems. In order to ensure the accuracy of the calculation results and save time and cost, model A without support is taken as an example to verify grid independence, and the average outlet velocity and pressure on the aortic wall are taken as evaluation indexes to ensure that other boundary conditions remain unchanged. Three sets of simulations with the number of grids of 195814, 297143, and 394247 were set up to verify the grid independence. The analysis results are shown in Table 1.

**Table 1:** Verification of grid independence of model A in fluid domain.

Number of grids (units)	Average exit velocity (m/s)	Average exit velocity Error (%)	Wall average Pressure value (MPa)	Wall average Pressure value error (%)
195814	0.1902	0	6.866	0
297143	0.1899	0.17	6.691	2.56
394247	0.1898	0.17	6.645	3.23

**Figure 2:**
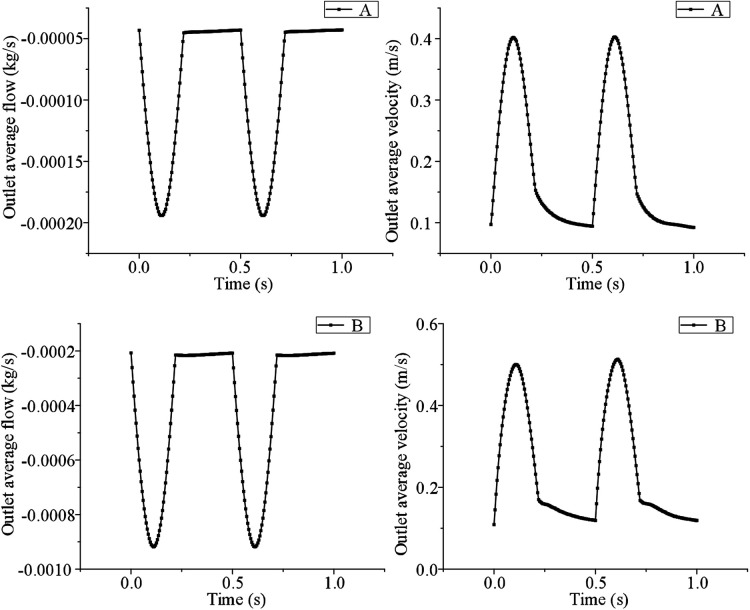
The simulation results were verified by grid independence and step independence between model A and model B.

The simulation results show that with the increase of grids, the average velocity and the average pressure error of the outlet gradually increase, but the error growth trend gradually decreases, and the error values are less than 5%. It can be seen that the increase in the number of grids has little influence on the simulation results, and when the number of fluid grids is 195,814, the requirements for the number and quality of grids in the simulation calculation can be met. Similarly, in order to eliminate the influence of the time step on numerical calculation results, it is necessary to carry out a step independence test. Taking model A without support as an example, the total time of numerical calculation is set to 1.5 s, and the time steps are set to 0.001 s, 0.0005 s, and 0.0001 s respectively. Taking the average outlet velocity and the average pressure on the main aortic wall as evaluation indexes, other boundary conditions are kept unchanged, the final error is guaranteed to be within 5%, and the numerical model with the time step of 0.001 s is selected for subsequent calculation. In the same way, the B model is verified by grid independence and step independence, and the simulation results are as follows (shown in Figure 2). We iterate coupling at each time step until the residual of the coupling system is less than the specified residual (25). In this study, the residual was set to 10^−6^. Hemodynamic characteristics were studied, including the blood flow field distribution, the blood pressure distribution, and the shear stress distribution on the wall of the LSA under half coverage.

What needs special emphasis is that this study uses CFD to simulate the hemodynamic changes after stent implantation, and the research object does not involve humans or animals, so ethical approval is not necessary.

## Results

### Velocity variation

#### Comparison of local blood flow velocity at the ostium of the LSA

**Figure 3:**
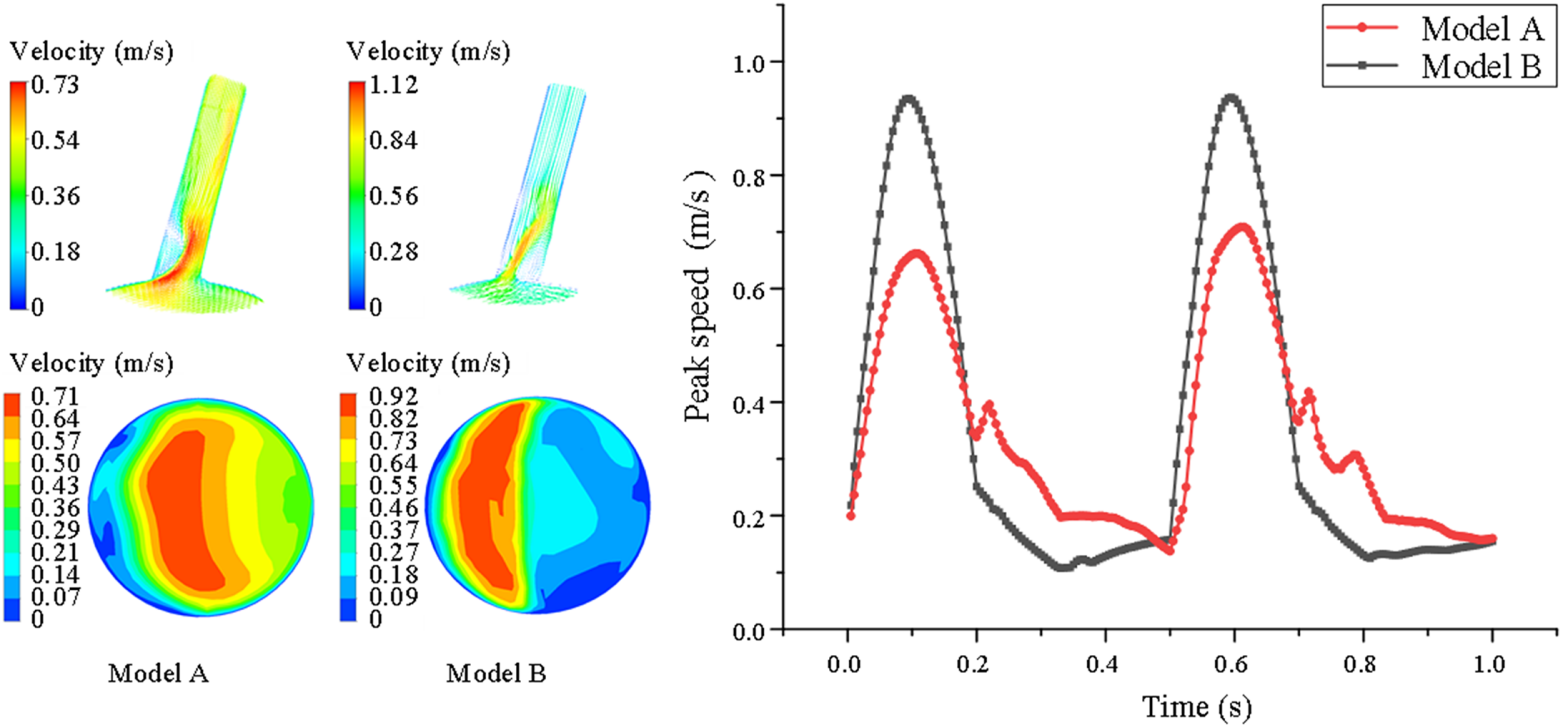
Comparison of velocity nephogram and local velocity nephogram between model A and model B. Meanwhile, the peak flow velocity changes in the whole cardiac cycle are also presented of the LSA in model A and model B. The darker the color, the higher the flow rate.

From the cross-sectional velocity nephogram of LSA, we intercept the peak time of aortic contraction to compare the local blood flow velocity at LSA ostium, we can see that the local blood flow velocity in model B is faster than model A. In addition, in the velocity nephogram of the local section of the LSA port, we can observe that Model A shows normal laminar flow, and the velocity in the central part is the fastest. However, due to the change in the blood flow direction of the LSA branch, a small part of the blood flow in the peripheral week became abnormal. However, in model B, most of the low-speed areas appear at the distal end of the stent covering the LSA ostium, while high-speed blood flow appears at the remaining ostium, and the local maximum blood-sucking flow velocity is significantly higher than that in model A (model A = 0.49 ± 0.12 vs. model B = 0.76 ± 0.24 m/s, *P* < 0.05) (shown in Figure 3).

#### Comparison of overall average velocity at the distal end of the LSA

**Figure 4:**
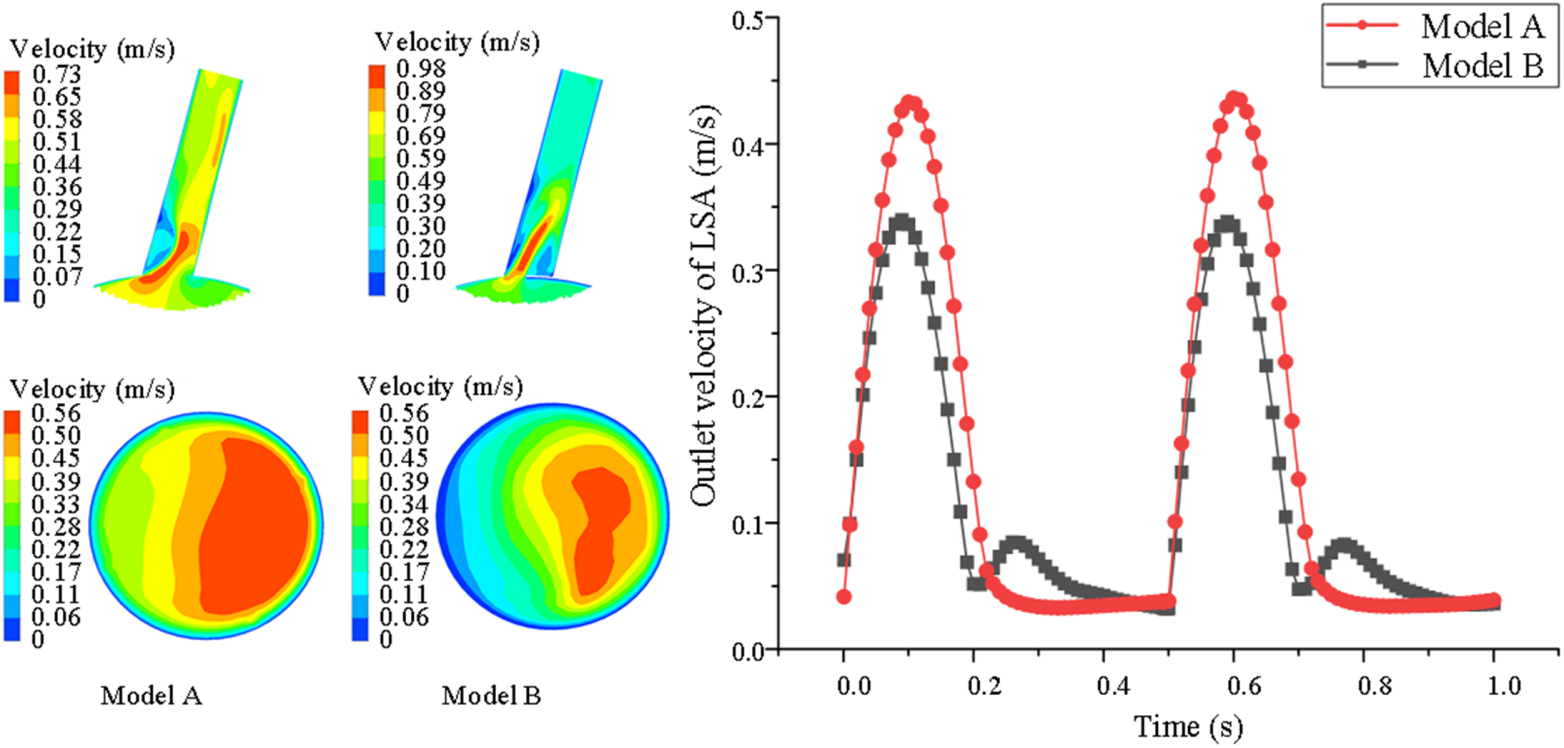
Comparison of velocity nephogram of blood flow and local lumen of LSA distal end in LSA lumen, aslo, changes of blood flow velocity of LSA with cardiac cycle are showed between model A and model B. The darker the color, the higher the flow rate.

However, for the overall average velocity at the distal end of the LSA, there were opposing results. The results show that this hemodynamic characteristic in model B was smaller than in model A. In model B, the LSA revealed the smallest range of high-speed blood flow at the distal end. In model A, the average velocity at the distal end was larger, because there was no stent interference (model A = 0.42 ± 0.16 vs. model B = 0.29 ± 0.11 m/s *P* < 0.05) (shown in Figure 4).

### Wall shear stress of LSA

**Figure 5:**
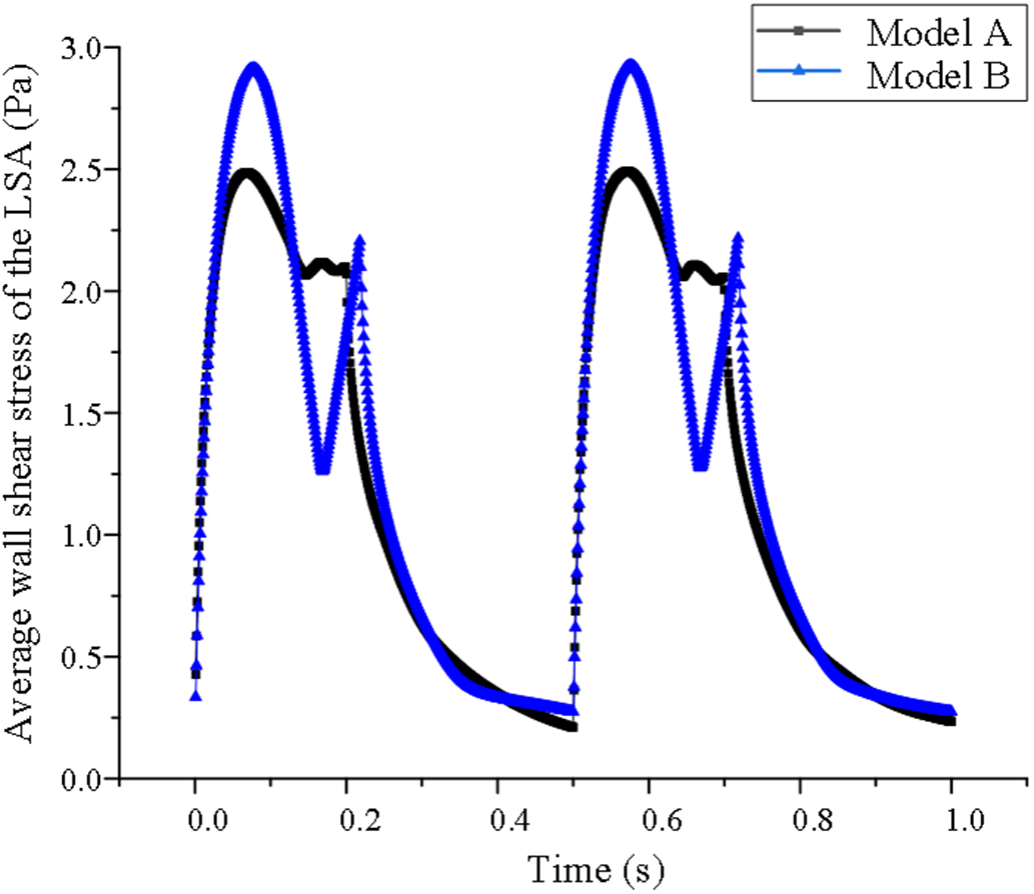
Average wall shear stress of the LSA in model A and model B.

Before stent implantation, the WSS of LSA in model A fluctuated regularly with the change in blood flow in cardiac cycle, indicating this indicator was related to blood flow velocity. During the cardiac cycle after stent implantation, the WSS of model B decreased in systole but did not change obviously in diastole, and showed a downward trend at the end of diastole. However, the average WSS is lower than that of Model A (1.13 ± 0.82 vs. 1.26 ± 0.92 Pa *P* < 0.05) (shown in Figure 5).

### The pressure change of the LSA

**Figure 6:**
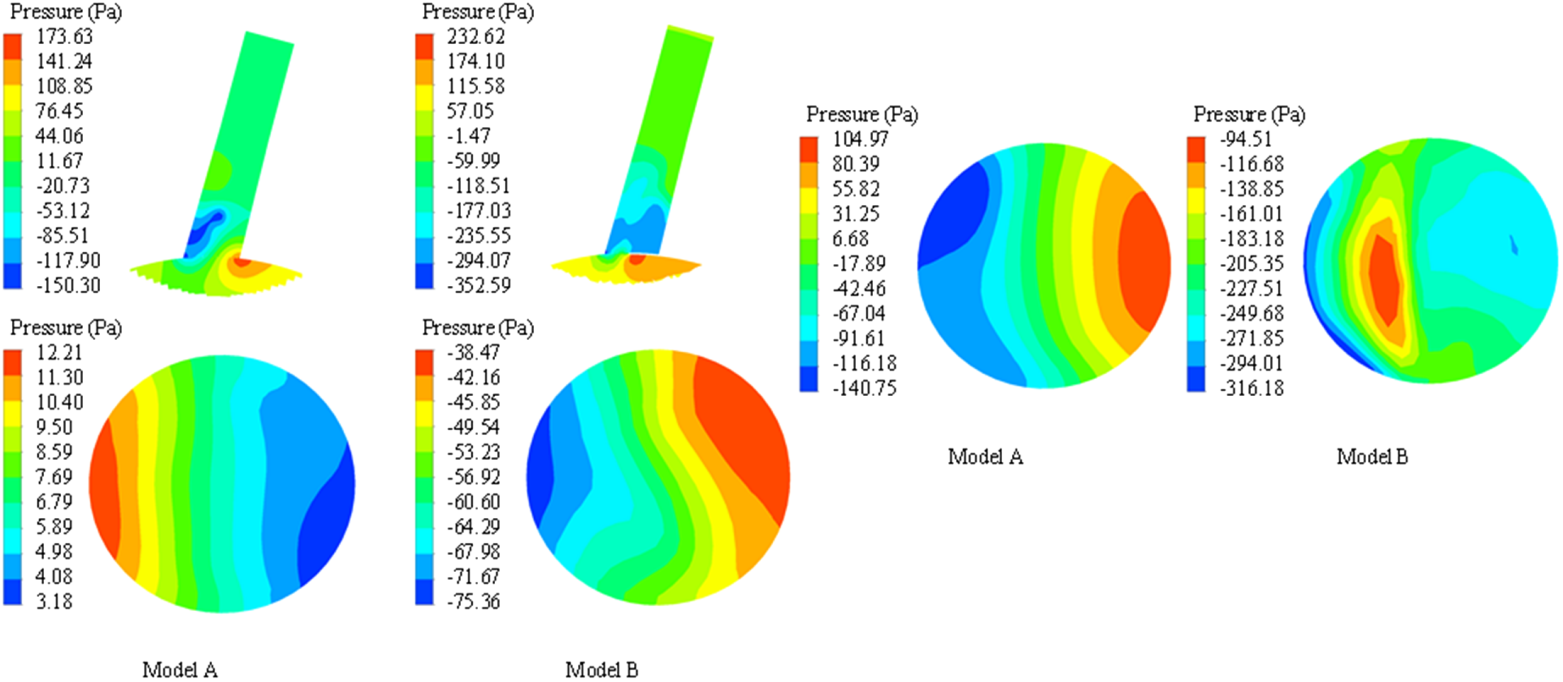
Comparison of pressure nephogram in longitudinal section, proximal ostium and distal end section of LSA between model A and model B. The darker the color, the higher the pressure.

Compared with the systolic peak, the local high pressure area of model A (before stent implantation) is located on the concave side of LSA, that is, the apex of arterial blood flow bifurcation, and the low pressure area is located on the convex side of LSA (shown in Figure 6A). In model B, the high pressure area is located at the proximal end of the endograft, which is the area where the stent graft causes blood flow bifurcation. In model B, the overall flow rate and velocity of LSA decreased due to the siphon effect of eccentric high-speed blood flow and the occlusion of the endograft, which caused the total pressure of the stent-covered LSA in the far proximal range to decrease and the relative low-pressure area to increase, but the low-pressure area at the far end may be reduced due to the impact of high-speed blood flow (shown in Figure 6B).

### Changes of blood flow state in LSA cavity

**Figure 7:**
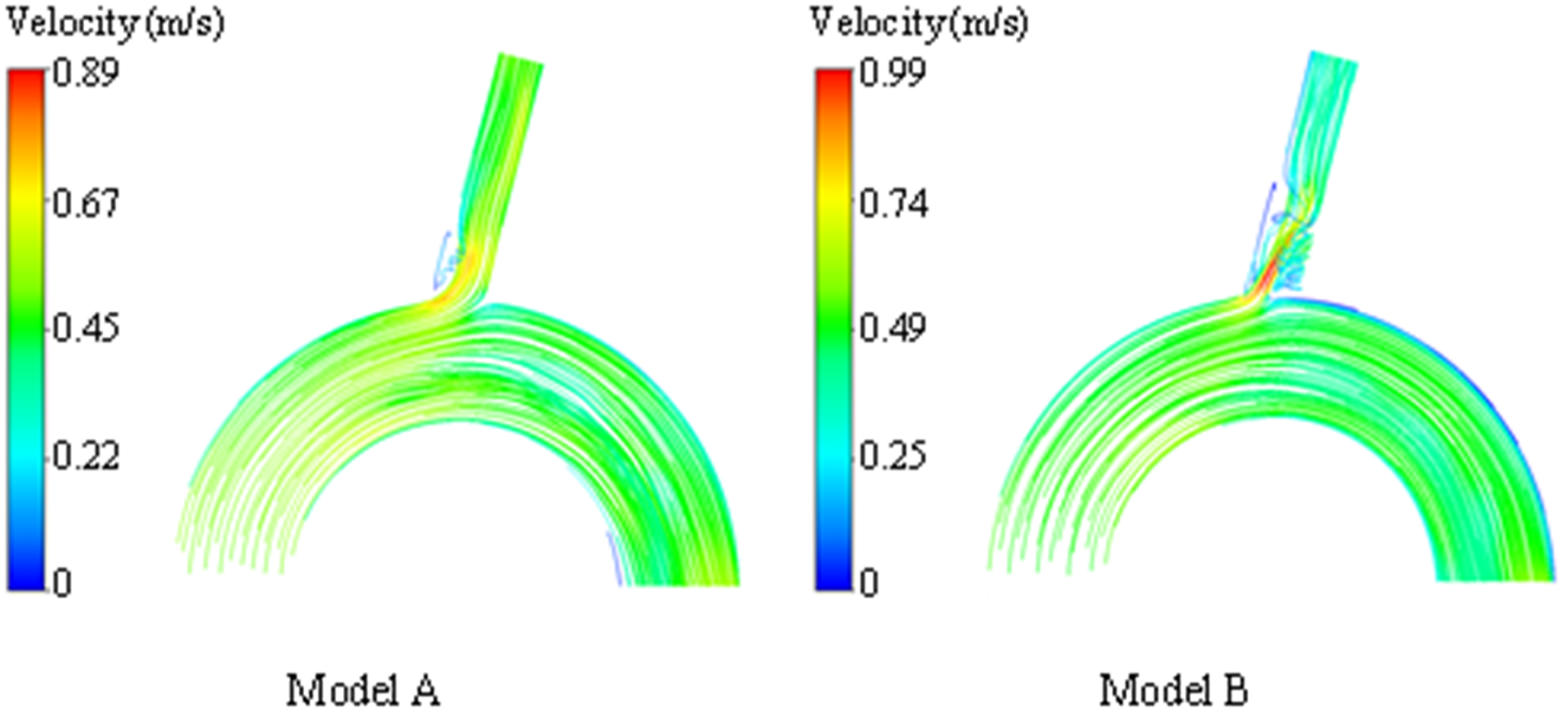
Streamline diagram of the systolic peak of the LSA in model A and model B.

Compared with model A, we can observe that the normal laminar flow at the far end of the LSA in model B becomes a low-speed vortex, and a large number of turbulent lines appear, which is due to the interference of the covered stent on the blood flow at the opening. This observation shows that in Model B after stent implantation, the flow pattern at the proximal end of LSA becomes disordered, especially when a large area of low-speed blood flow retention appears behind the stent film (shown in Figure 7).

## Discussion

TEVAR is developing rapidly, but it also faces many limitations (26). For example, in order to obtain sufficient PLA, diseases near LSA inevitably lead to stent implantation in Z2 area (involving the starting point of left subclavian artery, between left carotid artery and left subclavian artery), but partially or completely cover the LSA ostium, which may lead to claudication of the left upper arm or acute stroke. Therefore, the revascularization of LSA is needed (27) and many LSA reconstruction methods have been proceed, including surgical bypass, “chimney” technology, fenestration technology, single stent technology and so on (28). Although these revascularizations effectively preserve the blood perfusion of the LSA, they also changed the hemodynamics of LSA, which may cause or accelerate arteriosclerosis and thrombotic diseases (29). It is sometimes limited and time-consuming to observe the relationship between the formation of arteriosclerosis disease and hemodynamic changes in clinical research, which also need ethical issues (30). Therefore, it is very important to find an alternative, economical and effective method to study the changes of hemodynamics. At present, CFD is a research method that uses computer technology to build personalized or idealized models and simulate hemodynamics numerically, which is convenient, low cost, and time saving. It can quantify hemodynamic indexes, such as pressure distribution, wall shear stress and blood flow velocity, simulate the development trend of vascular system diseases before and after treatment, and is widely using in clinical research of these diseases (31). Therefore, the focus of our research is to observe and compare the hemodynamic effects of stent implantation in Z2 area covering half of the LSA ostium by CFD methods, and to predict the long-term effects on LSA.

Firstly, according to the theory of fluid mechanics, the low lumen flow rate and velocity of liquid will reduce the shear stress on the lumen wall (32), and WSS is a mechanical force exerted by blood flow on the surface of endothelial cells in the tangential direction, which is considered to be the most relevant mechanical factor for arteriosclerosis, stenosis and plaque rupture (33). Because low WSS increases the permeability of endothelial cells and interferes with the connection of arterial endothelial cells, so this process will weaken the barriers through which macromolecules pass, which leads to the increase of lipid uptake in atherosclerosis. At the same time, WSS is negatively correlated with the number of smooth muscle cells, however, exposure to normal laminar shear force environment will not affect the number of smooth muscle cells (34). From our research, it can be seen that the blood flow, average velocity and wall shear stress of LSA mouth are reduced due to semi-covering the LSA ostium, which is consistent with the above theory. Therefore, the hemodynamic changes caused by this surgical method will accelerate the arteriosclerosis process in the LSA in many ways, which is worthy of clinical study.

Secondly, blood can be regarded as a non-Newtonian fluid, only have axial motion (no lateral motion), which is called laminar flow. When the blood flow state change significantly, the laminar flow pattern begins to be destroyed and lateral movement perpendicular to the main flow direction occurs, thus forming turbulent flow. However, the blood flow velocity in the turbulent region is obviously slowed down, which is beneficial to the deposition of blood components, and reduce the tight connection between endothelial cells, which leads to lipid infiltration in the tube wall, finally cause inflammatory reaction and intimal hyperplasia. Therefore, it is considered that the turbulent flow mode has greater pressure on the pipe wall and is more likely to cause the formation of arteriosclerosis (35). From the velocity nephograms of the two models, it can be seen that after the laminar flow in the aorta flows into LSA, the direction and velocity of blood flow change sharply, and the fluid produces longitudinal and transverse velocities, and turbulence appears at the far side of blood flow bifurcation and the convex side of blood vessel curvature. At the same time, in model B, the semi-coverage of the LSA orifice by the internal graft will cause turbulent blood flow at the orifice, generate additional longitudinal vascular pressure, and eventually damage the vascular endothelium of the artery. Therefore, it is suggested from another aspect that the operation will accelerate the process of arteriosclerosis after changing the hemodynamics of the LSA.

Thirdly, when blood flows through the stenosis, it will form a downstream turbulent zone (36), and the local blood flow rate is extremely slow, which prolongs the contact time between blood flow components and the interface, thus promoting the aggregation of platelets, lipoproteins and other components relative to the wall, and finally leading to microthrombosis (37, 38). At the same time, the red blood cell volume in the low-speed turbulent region is also small, which is prone to local hypoxia, leading to the deposition of macromolecular substances, the increase of vascular wall permeability, and the injury of arterial intima. In our research, we have confirmed that half covering the LSA ostium decrease the distal velocity and format the local turbulence. Therefore, we have reason to predict that it will increase the possibility of arteriosclerosis in the LSA. On the other hand, the existence of turbulence and low velocity zone behind stent membrane may lead to local acute thrombosis. Therefore, it is worthy of our consideration whether patients undergoing this operation should receive routine antiplatelet therapy in clinic. Because, once a tiny thrombus is formed and falls off, it may cause serious vertebral base artery embolism.

### Limitations

Our work also has some important limitations. Firstly, it did not study the effects of different LSA angles and brachiocephalic trunk and left common carotid artery on hemodynamics and other indicators such as Max WSS, TAWSS, OSI, and RRT. Secondly, the boundary conditions were evenly set, which might not reflect the physiological and individual changes in the human body. We consider the main purpose of this study was to compare the hemodynamics changes of the special surgical methods on the left subclavian artery, so these limitations can be considered as a uniform control, and the influence of risk factors (such as hypertension) could be excluded. In addition, the numerical simulation experiment has its own limitations. It could only verify relevant results from the perspective of fluid flow patterns. The actual specific situation needs to be verified by animal experiments or long-term clinical follow-ups.

## Conclusion

In the past, for diseases near LSA, although some scholars tried to extend PLA by covering the LSA ostium with endograft, there was a lack of long-term follow-up data. Our CFD research results show that this surgical method greatly change the hemodynamics of the LSA and may increase the risk of arteriosclerosis and acute thrombosis. Therefore, routine Hypolipidemic therapy and antiplatelet therapy in patients without obvious contraindications after operation may have unexpected benefits.

## Data Availability

The datasets presented in this study can be found in online repositories. The names of the repository/repositories and accession number(s) can be found in the article/Supplementary Material.
